# How did COVID-19 pandemic impact on healthy behaviours among Polish professionally active physiotherapists aged 20–50?

**DOI:** 10.1186/s12889-024-19311-1

**Published:** 2024-07-09

**Authors:** Agnieszka Gołuchowska, Marta Balcerzak, Anna Lipert

**Affiliations:** 1 Department of Sports Medicine, Faculty of Health Sciences, Medical University of Lodz, Pomorska Street 251, Lodz, 92-213 Poland; 2 Military-Medical Faculty, Medical University of Lodz, Lodz, 90-647 Poland; 3 Department of Social and Preventive Medicine, Faculty of Health Sciences, Medical University of Lodz, Lodz, 90-752 Poland

**Keywords:** COVID-19 pandemic, Physiotherapists, Health behaviours

## Abstract

**Background:**

Due to the introduction of a number of changes to the health care system and the work nature of medical staff, theCOVID-19 pandemic still pose a public health challenge. The objective of the study was to characterize the health behaviours of Polish professionally active physiotherapists during the COVID-19 pandemic.

**Methods:**

The study included 104 Polish licensed and professionally active physiotherapists in whom health behaviours were assessed using an original questionnaire contained, among others, questions from the Global Physical Activity Questionnaire (GPAQ), the Pittsburgh Sleep Quality Index (PSQI) and the Perceived Stress Scale (PSS), available via social media platforms.

**Results:**

Among the physiotherapists, 34% worked directly with COVID-19 patients and 49% with those who had survived COVID-19. There were no statistically significant differences in most of the rates of physical activity undertaken by the physiotherapists surveyed (*P* > 0.05). Men were more likely to report taking up movement-related physical activity than women (*P* > 0.05). However, they spent more time sitting or lying down on a typical day (*P* > 0.05). The average time spent on the above-mentioned physical activities was also higher among the male participants than in the group of women (*P* > 0.05). There was an increase in the proportion of physiotherapists working over 40 h per week, from 29% before the pandemic to 38% during the pandemic. Statistically significant differences were observed for the products constituting the basison which of the diet of the examined physiotherapists was based (*P* < 0.05). The majority of the respondents reported no problems with falling asleep (*p* > 0.05). Stress related to the risk of contracting COVID-19, as well as concerns about the health of loved ones were more common and severe in the group of female subjects (*P* < 0.05).

**Conclusions:**

During the COVID-19 pandemic, the health behaviours in some physiotherapists changed. Further studies are required to assess whether physiotherapists’ health behaviours returned to baseline levels or slightly improved compared to the initial results. Also, it is necessary to introduce health-promoting initiatives that would focus on physiotherapists, support their positive health behaviours and provide special recommendations helping them to maintain health during a pandemic.

## Introduction

The epidemic state, resulting from the COVID-19 pandemic and declared by the World Health Organization (WHO) in March 2020, had an impact on the functioning of health systems around the world, leading to the development of new patterns of procedures and behaviours. The COVID-19 pandemic is the worst public health crisis of 21st century, and the necessity to reduce infections among healthcare workers and patients forced managerial staff to change the organization of operations. The global health emergency caused by the COVID-19 pandemic presents an unprecedented challenge for healthcare workers who have to deal with heavy workloads in mentally difficult situations.

The COVID-19 pandemic, becoming a stimulus for numerous restrictions, had a significant impact on the physiotherapy services subsector worldwide [1]. The working conditions of physiotherapists changed dramatically at that time due to, among others limited access to rehabilitation for non-infectious individuals, those with injuries, after surgery as well as chronically ill patients [2, 3]. As mentioned above, the restrictions imposed during the Covid-19 pandemic had a significant impact on reducing the availability of physiotherapy services which are an integral part of the healthcare system. This resulted from, among others, the requirement to comply with social distancing rules and temporal discontinuation of physiotherapy services in outpatient and home care. Thus, patients’ needs were clearly affected during this period. The reorganization of existing methods and standards of practice required urgent changes in the work of physiotherapists [4], who were forced to look for new solutions, taking into account the control requirements [5].

Another limitation reported by many physiotherapists around the world was occurrence of burnout and psychological problems in this professional group, which resulted from stress and effort related to work in pandemic conditions [6].

The COVID-19 disease, its course, duration and short- and long-term health consequences significantly increased requirements for rehabilitation services [4, 7]. A specific interdisciplinary and personalised physiotherapeutic approach [8] became necessary for patients recovering from COVID-19, people in whom the disease has developed into a new disease, i.e. long COVID, patients with comorbidities, disabled patients, as well as those who experienced serious post-COVID complications [9, 10].

The main task of physiotherapists was to support patients after COVID-19 in returning to normal functionality, professional activity, regaining independence, minimizing negative consequences of illness, reducing disability, being a common complication [11, 12], as well as alleviating psychological problems [13].

In the face of the global health threat caused by COVID-19, medical staff, including physiotherapists, as a high-risk group and a major link in ensuring health safety to patients, have been exposed to many changes in their professional duties and lifestyle. Therefore, monitoring of health behaviours among medical staff is a key element of public health activities aimed at lessening the negative impact of the pandemic, also in the future.

Recent literature lacks comprehensive reports on characteristics of health behaviours in professionally active physiotherapists during the COVID-19 pandemic, therefore the authors have decided to present the findings from a more detailed analysis conducted among this group of healthcare professionals.

The available publications mainly provide information on the parameters of the health status of medical professionals, in particular mental health, and interventions that promote a wide range of their activities [14, 15], as well as conditions of support for medical staff cooperating with patients during the COVID-19 pandemic and affected by its impact [16, 17]. The literature shows that among medical staff, most attention wasfocused on nurses [18, 19].

The literature provides little information on performance and working conditions of physiotherapists during the COVID-19 pandemic or their health behaviour changes resulting from the introduced pandemic restrictions [20, 21]. Hence the purpose of our study was to investigate which general health behaviours were characteristic for professionally active physiotherapists during the COVID-19 pandemic/to characterise health behaviours of professionally active physiotherapists during the COVID 19 pandemic.

## Methods

### Study design and participants

The study was a diagnostic survey and included 104 Polish licensed and professionally active physical therapists, both men and women. The main inclusion criteria were: 1) age between 18 and 65 years;2) holding the right to practise the profession of a physiotherapist; 3) active professional work during the COVID-19 pandemic period; 4) voluntary consent to participate in the study; 5) submission of a signed form with written informed consent. Exclusion criteria for the sample were the following: age below 18 and over 65 years, lack of active work during the pandemic period or lack of the license to practise the profession of a physiotherapist. The sample size was calculated based on the results of a pilot study and assuming 20% non-response. The questionnaire was filled out incorrectly in 28 cases. Most often, those irregularities were due to failure to meet either the criterion of inclusion in the study or any of the exclusion criteria, or misunderstanding.

The Ethics Committee at the Medical University of Lodz (Poland) decided to waive the ethical approval and the requirement for informed consent. The Committee does not evaluate studies that are not considered medical experiments (e.g. biochemical tests, pharmaceutical tests, etc.), and are conducted without the use of material collected from patients.

Therefore, neither ethical approval nor consent to participate in the study were required. By completing the survey questionnaire, the participants agreed to take part in the study. Participation in the survey did not involve disclosure of any personal data, and all the answers are anonymous.

The study included 104 Polish licensed physical therapists, both men and women, and the main inclusion criterion was having the right to practise the profession of physiotherapist and active work during the COVID-19 pandemic period.

### Procedure

In order to characterise working conditions of physiotherapists participating in the study and their most important lifestyle determinants during the COVID-19 pandemic, an original diagnostic survey was used. it was based on the previously validated questionnaires such as the Global Physical Activity Questionnaire (GPAQ) [22–24], the Pittsburgh Sleep Quality Index (PSQI) [25] and the Perceived Stress Scale (PSS) [26]. There were also several additional questions related to eating habits, stimulants and available on-line via web platforms and disseminated in groups of physiotherapists on social media, with the prior consent obtained from the relevant moderators. The questionnaire included anthropometric and sociodemographic information: the sex, age, weight and height of the study participants. It contained 49 specific questions, including 11 open-ended ones, 34 closed single-choice questions and 4 multiple-choice questions with an option of adding a respondent’s own answer/ mostly closed and semi-open ones. The survey was available on-line via web platforms and disseminated in groups of physiotherapists on social media, with the prior consent obtained from the relevant moderators. A total of 132 questionnaires were filled in, out of which 104 were deemed valid, whereas 28 were considered invalid because of incomplete answers or incorrect entered values. This yielded an effective response rate of 78.79%.

Based on the obtained data, Body Mass Index (BMI) was calculated for each subject using the formula weight (kg)/height (m)2. The physiotherapists referred to their working conditions and health behaviours including physical activity, diet, stimulants used, quality and quantity of sleep, as well as perceived stress level experienced during the COVID-19 pandemic.

### Applied instruments

Ten questions from the Polish version of the Global Physical Activity Questionnaire (GPAQ) [22] were used to assess the level of physical activity. The sections related to mobility, and recreational and sedentary activities from this version of the questionnaire were used in the study [22]. Activity with a flow rate of 4 MET was assumed to be moderate intensity, and 8 MET to be high intensity [22]. The reliability of the GPAQ was initially assessed in nine countries and was standardised to be used as an international research tool regardless of a geographic region or socio-cultural and economic characteristics of the population [23]. The GPAQ-A obtained an internal consistency of Cronbach’s alpha 0.93 [24].

For subjective assessment of the level of stress, an original six-point numerical scale was applied, where 0 meant no stress, and 5 corresponded to severe, paralysing stress. Ten questions were used from PSS which is a validated and classic stress assessment instrument whose internal reliability determined using the Cronbach`s alpha ranges from 0.84 to 0.86 [25].

The quality and patterns of sleep for adults were measured using the questions from the PSQI which has strong reliability and validity in a variety of samples, suggesting that the tool fulfils its intended utility. The PSQI questionnaire is characterized by high internal consistency, with Cronbach’s alpha index of 0.83 [26]. The data were collected between 7 October 2021 and 22 February 2022.

### Statistical analysis

The statistical analysis was performed with the Statistica software (13.3 Stat Soft). The values were expressed as the arithmetic mean (𝑥) and standard deviation (SD). Normality of distribution was tested with the Shapiro–Wilk test. Due to the fact that the variables were not normally distributed, to analyse the differences between the groups, the U Mann–Whitney and Spearman’s rank correlation coefficient were used. The correlation condition is a value of the Spearman rank coefficient equal to or greater than 0.2. To compare the results obtained in the groups of females and males with the total studied population, the structure index of all the respondents (%O) was applied. The difference was considered statistically significant when the p value was lower than 0.05.

## Results

The study group comprised 104 Polish physiotherapists, including 74 females (aged 23–46 years, mean age = 29.77 ± 4.9 years) and 30 males (aged 23–43 years, mean age 30.2 ± 5.1 years). The findings indicate comparability of the analysed groups due to the lack of statistical differences in terms of age, educational level, seniority and average weekly working time before and during the COVID-19 pandemic (*p* > 0.05). There were statistically significant differences in terms of body weight and BMI values between the groups of the respondents (*p* < 0.05).

The results show that most physiotherapists (66%) did not work with COVID-19 patients. The groups of physiotherapists did not differ in the frequency of rehabilitation of patients after COVID-19, however, this form of therapy was more often performed by female than male physiotherapists than males (53% vs. 40%, respectively; *p* > 0.05).

Women most often worked in rehabilitation clinics (36%), private offices (36%), and home rehabilitation (26%), while men in rehabilitation clinics (43%), sports clubs, gyms and fitness clubs (37%), and in private offices (33%).

The characteristics of the examined physiotherapists, including their professional activity, are presented in Table 1.

**Table 1:** Characteristics of all the physiotherapists included in the study

Variable	Females *N* = 71	Males *N* = 30
	Mean (± SD)	
Age [years]	29.77 ± 4.9	30.2 ± 5.1
Height [kg]	65.9 ± 11.7*	85.8 ± 10.7
BMI [kg/m^2^]	23.21 ± 4.3*	25.9 ± 2.5
	**N (%)**	
**Education**		
Technician or Bachelor’s degree in Physiotherapy	6 (8)	4 (13)
Master’s degree in Physiotherapy	66 (88)	26 (86)
Doctor of Physiotherapy	2 (3)	0
**Work experience in the profession of physiotherapist**		
Under 5 years	38 (51)	11 (37)
5–10 years	24 (32)	13 (43)
11–15 years	7 (9)	4 (13).
16–20 years	4 (5)	2 (7)
21–25 years	1 (3)	0
Over 25 years	0	0
Place of work **		
Rehabilitation clinic/centre	27 (36)	13 (43)
Private office	27 (36)	10 (33)
Hospital	8 (11)	2 (7)
Nursing home or health care centre	5 (7)	0
Sanatorium or health restoration hospital	1 (1)	2 (7)
Individual physiotherapy practice	19 (26)	4 (13)
Sports club / gym / fitness club	0 (0)	11 (37)
Massage room / SPA room	7 (9)	2 (7)
Other	10 (14)	1 (3)
**Average weekly working time before the COVID-19 pandemic (until March 2020)**		
Less than 10 h	6 (8)	2 (7)
11–25 h	3 (3)	0
26–35 h	11 (16)	3 (10)
36–45 h	40 (54)	18 (60)
46–55 h	9 (12)	6 (20)
56–65 h	3 (4)	0
Over 66 h	2 (2)	1 (3)
**Average weekly working time during the COVID 19 pandemic**		
< 10 h	1 (1)	0
11–25 h	8 (11)	0
26–35 h	13 (19)	5 (17)
36–45 h	36 (49)	16 (53)
46–55 h	12 (16)	6 (20)
56-65 ours	2 (2)	2 (7)
> 66 h	2 (2)	1 (3)
**Active work with COVID-19 patients**		
Yes	25 (34)	10 (33)
No	49 (66)	20 (67)
**Motor rehabilitation of patients after COVID-19**		
Yes	39 (53)	12 (40)
No	35 (47)	18 (60)

* Significant difference if *p* < 0.05

### Characteristics of the physical activity level in the study group of physiotherapists during the COVID-19 pandemic

According to the answers given by the physiotherapists, 84% of the survey participants spent at least ten minutes a day taking up physical activity involving mobility, such as walking and/or cycling.

There were no statistically significant differences in most rates of physical activity undertaken by the physiotherapists surveyed (*P* > 0.05). Men were more likely to report taking up movement-related physical activity, and moderate to high-intensity activities (*P* > 0.05) than women. However, they spent more time sitting or lying down on a typical day (*P* > 0.05). The average time spent on the above-mentioned physical activities was also higher than in the group of women (*P* > 0.05).

It was found that nearly one third of all the participants (*n* = 32, 31%) walked and/or cycled for at least 10 min daily, without a break. However, 11% of the respondents did not devote a single day a week to such an activity. When analysing the relationship between gender and the number of days devoted to movement-related activity, no correlation was identified (R Spearman = 0.14, *p* = 0.14). It was noted that in total, the respondents spent an average of 185 min per week walking and/or cycling, and they undertook these forms of physical activity three to four days a week averagely. Women spent an average of 50.5 min per day walking and/or cycling, and men spent an average of 64.2 min (median for both men and women was 30 min). There was no correlation between gender and time spent on movement-related physical activity (R Spearman=-0.03, *P* = 0.7).

The vast majority (63%) of the surveyed physiotherapists engaged in high-intensity recreational activities (e.g., running, playing football, etc.), resulting in a significant increase in breathing rate and heartbeat. However, the study findings indicate no correlation between the respondents’ gender and undertaking the above-mentioned type of activity (R Spearman=-0.17, *P* = 0.08).

Analysis of the collected data shows that women devoted an average of 31.6 min to high-intensity physical activity on a typical day, and men 66.7 min. In total, the respondents devoted an average of 67 min per week to high-intensity physical activity and undertook this form of activity on average 1.6 days a week. There was no correlation between gender and duration of activity on a typical day (R Spearman = 0.18).

The largest percentage of the participants (62%) were people undertaking recreational activity of moderate intensity at least once a week, lasting minimum ten minutes without a break. Statistical analysis of the obtained data indicates no correlation between the respondents’ gender and moderate-intensity activity (R Spearman=-0.05, *P* > 0.05).

Among the overall group of physiotherapists studied, 27% undertook recreational activity of moderate intensity two times a week, thus constituting the largest group among individuals practising sports. There was no correlation between gender and the number of days devoted to recreational activities resulting in mildly accelerated breathing rate and heartbeat (R Spearman = 0.11, *P* > 0.05).

Despite the difference in the mean duration of moderate-intensity physical activity between the groups of women (mean 32 min) and men (mean 39.3 min), the median for both groups was the same at 30 min (R Spearman = 0.05, *P* > 0.05). Overall, the respondents devoted an average of 55 min per week to moderate-intensity recreational activities, and undertook this form of activity 1.6 days a week, on average.

According to the results of the study, women spent less time sitting or lying down on a typical day (an average of 2.9 h in the women’s group and 3.3 h in the men’s group). The results indicate no linear correlation between sex and time spent sitting or lying down on a typical day (R Spearman − 0.1, *P* = 0.3).

Table 2 presents the characteristics of the level of physical activity of the participants.

**Table 2:** Characteristics of the physical activity level among the surveyed physiotherapists based on the global physical activity Questionnaire GPAQ (Polish version)

Variable	Females *N* = 74	Males *N* = 30
	*N* (%)	
**Walking/Cycling for at least 10 min continuously during a typical week**		
Yes	60 (81)	27* (90%)
No	14 (19)	3* (10%)
**Walking/Cycling for at least 10 min continuously (days/week)**		
0 days	9 (12)	2 (7)
1 days	3 (4)	0
2 days	6 (8)	1 (3)
3 days	13 (18)	6 (20)
4 days	9 (12)	3 (10)
5 days	10 (14)	4 (13)
6 days	3 (4)	3 (10)
7 days	21 (28)	11 (37)
**Moderate-intensity recreational activity for at least 10 min during a typical week**		
Yes	38 (51)	27 (90)
No	36 (49)	3 (10)
**Moderate-intensity recreational activities (days/week)**		
0 days	28 (38)	9 (30)
1 day	11 (15)	4 (13)
2 days	20 (27)	8 (27)
3 days	9 (12)	4 (13)
4 days	3 (4)	0
5 days	2 (3)	1 (3)
6 days	0	2 (7)
7 days	1 (1)	2 (7)
**High-intensity recreational activities for at least 10 min during a typical week**		
Yes	43 (58)	23 (77%)
No	31 (42)	7 (23%)
**High-intensity recreational activities (days/week)**		
0 days	28 (38)	7 (23)
1 day	14 (19)	5 (17)
2 days	14 (19)	7 (23)
3 days	14 (19)	5 (17)
4 days	2 (3)	5 (17)
5 days	1 (1)	0
6 days	0	1 (3)
7 days	1 (1)	0
	Mean (± SD)	
**Time spent sitting or lying down [hours/day]**	2,9 ± 2,3*	3,3 ± 4,3
**Time spent walking or cycling [minutes/day]**	50,5 ± 79,9*	64,2 ± 115,3
**Time spent on moderate-intensity physical activity [minutes/day]**	32 ± 38,2*	39,3 ± 56,4
**Duration of high-intensity physical activity [minutes/day]**	31.6 ± 32.2*	66.7 ± 88.4

* Significant difference if *p* < 0.05

### Characteristics of the diet followed by the study group of physiotherapists during the COVID-19 pandemic

During the COVID-19 pandemic, physiotherapists most often ate three to five meals a day (34% of women vs. 30% of men). There was no correlation between gender and the number of meals consumed (R Spearman = 0.007, *P* = 0.9). Among the physiotherapists surveyed, 26% had never snacked between meals. When analysing the relationship between sex and snacking, there was no correlation (R Spearman = 0.14, *P* = 0.16), which suggests that both women and men have snacks between meals. It was shown that the majority of the respondents (39%) did not eat regularly, and there was no relationship between gender and regularity in eating meals in the study groups (R Spearman=-0.06).

The analysis of the collected data shows that there is a positive correlation between sex and consumption of whole grains during the pandemic (R Spearman = 0.3, *P* = 0.002), fruit consumption (R Spearman = 0.3, *P* = 0.001) and consumption of sweets (R Spearman = 0.2, *P* = 0.04).

There was no correlation between gender and change in dairy intake (R Spearman = 0.16, *P* = 0.11), or vegetable consumption (R Spearman = 0.15, *P* = 0.13) and fast food consumption (R Spearman = 0.06, *P* = 0.5) among the groups of the physiotherapists. The diet of the majority of the women surveyed was based on cereal products (38%), whereas in the case of the most of the men, it was (63%).

It was noted that among all beverages, water was the most frequently consumed during the day (63% of the whole group). The second most frequently chosen drink was tea (19%).

The vast majority of the respondents (54%) claimed that they had not changed their diet during the pandemic.

According to the respondents, 30% of women and 17% of men improved their diet. However, there was no correlation between gender and change in the diet of the surveyed physiotherapists during the pandemic (R Spearman=- 0.14, *P* = 0.17). No differences were observed between the study groups in terms of the number of meals consumed, regularity of their consumption or snacking between meals (*P* > 0.05).

The physiotherapists were similar in terms of their consumption of cereal products, fruits and sweets, confectionery and salty snacks (*P* < 0.05). They did not differ in the consumption of dairy products, vegetables or fast food products (*P* > 0.05).

Statistically significant differences were observed for the products constituting the basis of the diet of the examined physiotherapists (*P* < 0.05). Among women those were most often grain products (38%), while among men, it was meat (64%).

Additionally, physiotherapists were similar in terms of beverage consumption and subjective assessment of dietary change during the pandemic (*P* > 0.05).

**Fig. 1:**
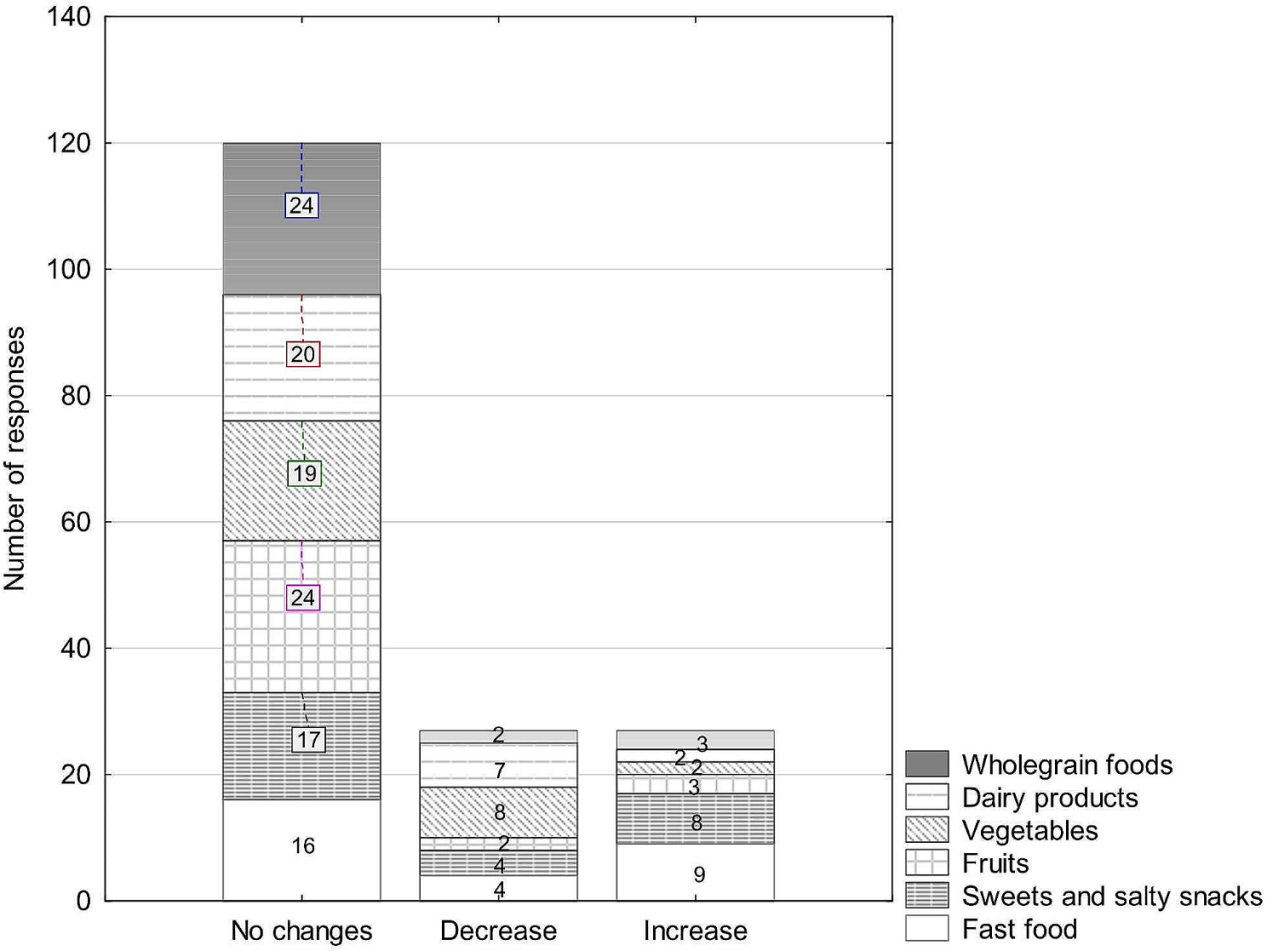
Changes in the consumption of products due to the COVID − 19 pandemic observed in men

**Fig. 2:**
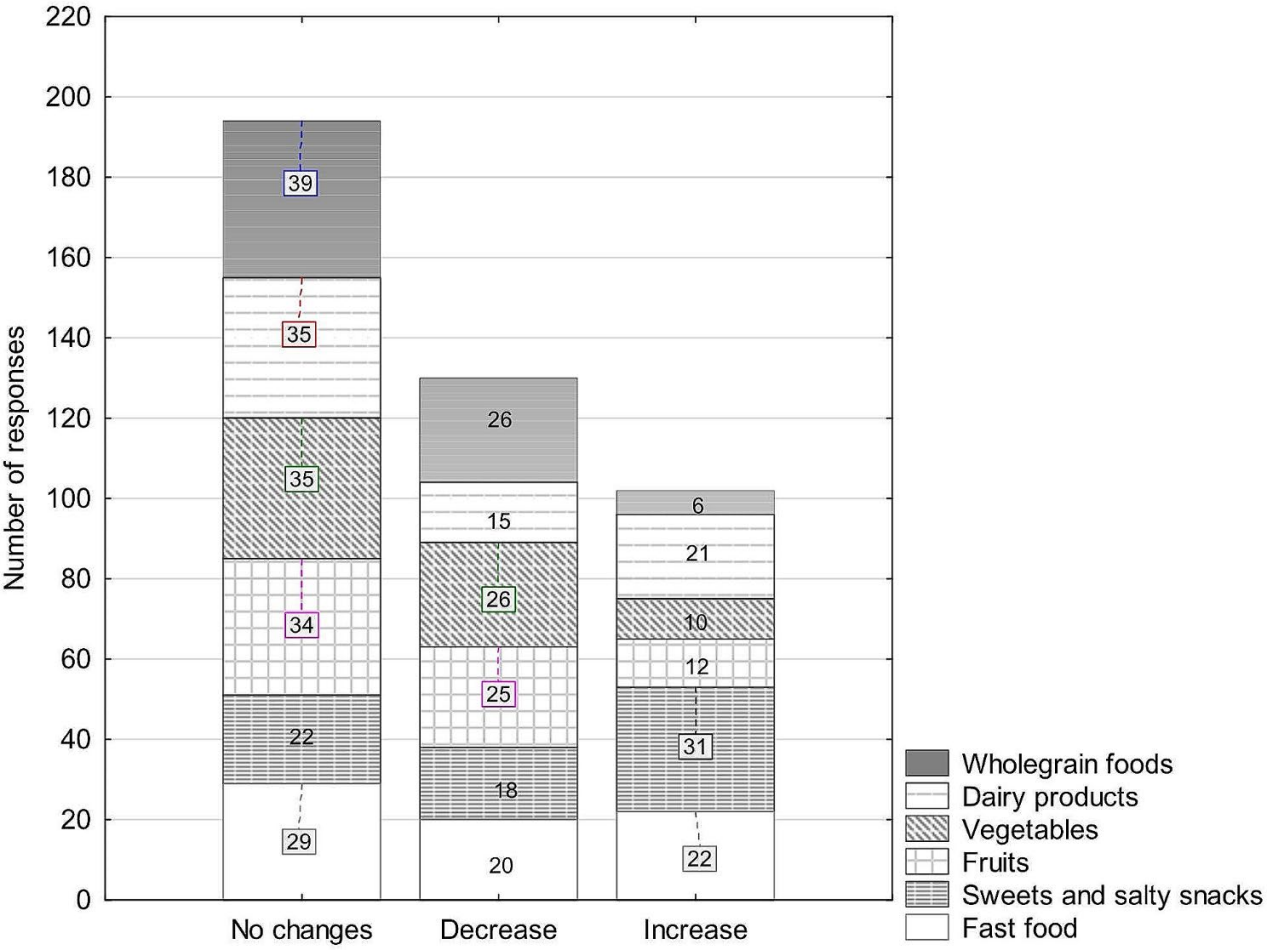
Changes in the consumption of products due to the COVID − 19 pandemic observed in women

Table 3 presents the characteristics of the diet of the surveyed physiotherapists during the COVID-19 pandemic. Figures 1 and 2 present the changes in the consumption of products due to the COVID-19 pandemic observed in men and women.

**Table 3:** Subjective characteristics of nutrition during the COVID-19 epidemic among the surveyed physiotherapists

Variable	Females *N* = 74	Males *N* = 30
	*N* (%)	
**Number of meals consumed per day**		
one meal a day	0	0
two meals a day	2 (3)	3 (10)
three meals a day	24 (32)	9 (30)
four meals a day	25 (34)	8 (27)
five meals a day	19 (27)	8 (27)
six or more meals	3 (4)	2 (6)
**Eating meals at regular times**		
Yes	23 (31)	12 (40)
No	31 (42)	10 (33)
Sometimes	20 (27)	8 (27)
**Snacking between meals**		
Yes	28 (38)	8 (27)
No	20 (27)	7 (23)
Sometimes	26 (35)	15 (50)
**Most consumed foods on a typical day**		
Grain products	28 (38)	4 (13)
Dairy products (excluding meat)	10 (14)	3 (10)
Vegetables	23 (30)	4 (13)
Fruit	3 (4)	0
Meat	10 (14)	19 (64)
**Most consumed beverages on a typical day**		
Water	45 (60)	21 (71)
Tee	17 (23)	3 (10)
Coffee	10 (14)	4 (13)
Flavoured water	0	0
Carbonated drinks	0	1 (3)
Fruit juices and nectars	2 (3)	1 (3)
**Subjective assessment of nutrition during the COVID-19 pandemic**		
Definitely better than before the pandemic	7 (9)	1 (3)
Better than before the pandemic	15 (20)	4 (13)
Same as before the pandemic	39 (53)	17 (57)
Worse than before the pandemic	8 (11)	8 (27)
Definitely worse than before the pandemic	5 (7)	0

* Significant difference if *p* < 0.05

### Characteristics of stimulant consumption in the study group of physiotherapists during the COVID 19 pandemic

During the pandemic, 14% of all the physiotherapists surveyed smoked cigarettes (after a break or for the first time in their lives), and 6% smoked more than before. An increase in the amount of cigarettes smoked during the pandemic was declared by 17% of the men surveyed.

Alcohol, on the other hand, was more often used by 16% of the respondents, whereas 22% of the study subjects reduced its consumption. Also, 15% of women and 20% of men admitted they had drunk alcoholic beverages more often.

During the pandemic, 22% of all the respondents consumed coffee and energy drinks more often (22% of women vs. 23% of men, *P* > 0.05), and 9% of the respondents (8% of women and 10% of men) began using sedatives.

Table 4 presents the characteristics of consumption of stimulants in the study group during the COVID-19 pandemic.

**Table 4:** Characteristics of stimulant consumption in the study group during the COVID 19 pandemic

Variable	Females *N* = 74	Males *N* = 30
	*N* (%)	
I quit smoking	5 (7)	3 (10)
I started smoking	3 (4)	1 (3)
I smoke more than before the pandemic	1 (1)	5 (17)
I drink alcohol less often	16 (22)	7 (23)
I drink alcohol more often	11 (15)	6 (20)
I drink less coffee and energy drinks	2 (3)	2 (7)
I drink more coffee and energy drinks	16 (22)	7 (23)
I have no opinion	39 (53)	12 (40)
**Did you start taking sedatives during the COVID-19 pandemic?**		
Yes	6 (8)	3 (10)
No	68 (92)	27 (90)

* Significant difference if *p* < 0.05

### Quantitative and qualitative characteristics of sleep in the study group of physiotherapists during the COVID-19 pandemic

During the Covid-19 pandemic, 54% of the respondents slept from seven to eight hours daily. Over one third of the respondents (40%) slept less than seven hours a day. More than half of the respondents (56%) slept from seven to eight hours at weekends, whereas nearly one third (32%) from nine to ten hours a day. There was no correlation between gender and sleeping time on weekdays (Spearman *R*=-0.017) and at weekends (Spearman *R* = 0.08).

The majority of the respondents (66% of the study group) reported no problems with falling asleep (*p* > 0.05). However, there was no relationship between gender and the occurrence of problems with falling asleep (R Spearman=- 0.09, *P* = 0.4), or between sex and waking up at the same time (R Spearman = 0.17, *P* = 0.08).

The results show that 50% of the respondents woke up at the same time every morning and 43% at night. There were statistically significant differences in declarations concerning waking up at night and waking up before the alarm clock (*p* < 0.05). Both occurrences were more common in the group of women. There was a statistically significant correlation (R Spearman = 0.3, *P* = 0.02) between gender and waking up before the alarm clock (R Spearman = 0.2, *P* = 0.046). According to the results, 17% of the respondents confirmed that they had suffered from sleep disorders during the pandemic. However, no correlation was found between gender and the presence of this indicator in the group of physiotherapists studied (R Spearman = 0.12, *P* = 0.2).

Table 5 presents the quantitative and qualitative characteristics of sleep in the studied group of physiotherapists during the COVID-19 pandemic.

**Table 5:** Quantitative and qualitative characteristics of sleep among the study participants

Variable	Females *N* = 74	Males *N* = 30
	*N* (%)	
**Number of hours of sleep per day (Monday – Friday)**		
< 5 h	1 (1)	1 (3)
5–6 h	30 (42)	10 (33)
7–8 h	38 (51)	18 (61)
9–10 h	4 (5)	1 (3)
> 10 h	1 (1)	0
**Number of hours of sleep (Saturday-Sunday)**		
< 5 h	1 (1)	0
5–6 h	7 (9)	2 (7)
7–8 h	37 (50)	21 (70)
9–10 h	28 (39)	5 (16)
> 10 h	1 (1)	2 (7)
**Problems with falling asleep**		
Yes	10 (14)	2 (7)
No	44 (59)	25 (83)
Sometimes	20 (27)	3 (10)
**Waking up at the same time every day**		
Yes	41 (55)	11 (37)
No	33 (45)	19 (63)
**Waking up at night**		
Yes	39* (53)	6 (20)
No	35* (47)	24 (80)
**Waking up before the alarm clock**		
Yes	30* (41)	6 (20)
No	44* (59)	24 (80)
**Sleep problems/Worsening of previous sleep problems during the COVID-19 pandemic**		
Yes	15 (20)	3 (10)
No	59 (80)	27 (90)

* Significant difference if *p* < 0.05

### Characteristics of the level of stress and anxiety in the study group of physiotherapists during the Covid-19 pandemic

Stress related to the risk of contracting COVID-19, as well as concerns about the health of loved ones were more common and severe in the group of female subjects (*P* < 0.05). The results indicate statistically significant strong correlations between the gender of the respondents and the feeling of anxiety about the health of loved ones in the last 12 months (R Spearman = 0.6, *P* = 0.31), as well the level of anxiety experienced (R Spearman=- 0.5, *P* = 0.6).

Table 6 shows the subjective assessment of the level of stress among the surveyed physiotherapists.

**Table 6:** Subjective assessment of the level of stress among the surveyed physiotherapists

Variable	Females *N* = 74	Males *N* = 30
	*N* (%)	
**Feeling stress caused by the risk of contracting COVID-19 in the last 12 months**		
Yes	49* (66)	9 (30%)
No	25* (34)	21 (70%)
**Level of perceived stress caused by the risk of contracting COVID-19 (0-**,** 5 - severe**,** paralysing stress)**		
0	21* (28)	19 (64)
1	6 (8)	4 (13)
2	16* (22)	1 (3)
3	17* (23)	3 (10)
4	12 (16)	3 (10)
5	2 (3)	0
**Level of perceived stress caused by the risk of contracting COVID-19**		
0	5* (7)	18 (58)
1	4 (6)	1 (3)
2	7 (9)	4 (13)
3	20* (27)	3 (10)
4	23* (31)	4 (7)
5	15* (20)	0
**Feeling stress related to the risk of losing your job in the last 12 months**		
Yes	25 (34)	11 (37%)
No	49 (66)	19 (63%)
**Declared ways of dealing with stress during the COVID-19 pandemic**		
Reading/listening to music/watching movies	39 (53)	16 (53)
Meditation/relaxation techniques	25* (34)	10 (33)
Conversation	41 (55)	8 (27)
Physical training	36 (49)	17 (57)
Other	10 (14)	5 (17)

* Significant difference if *p* < 0.05

## Discussion

The COVID-19 pandemic posed a lot of new challenges to medical staff. The mode of work changed and, as a consequence, often the location of the workplace as well. Social distancing and enforced isolation contributed to rapid lifestyle changes, significantly affecting the health of the population.

The experience of the pandemic showed a greater recognition of physiotherapeutic services [13]. The actions taken by physiotherapists, trying to meet the specific challenges of the pandemic, brought about certain changes in their health behaviours. We attempted to characterise health/lifestyle behaviours among professionally active physiotherapists during the COVID-19 pandemic, and our study found that in the face of the virus, these behaviours had changed.

According to the data of 30 March 2020 presented by the Polish National Chamber of Physiotherapists, approximately 75% of physiotherapists (50.000 people) were forced to suspend their professional activities in the Republic of Poland due to the COVID-19 pandemic [27]. Similar trends were observed in the German health service. According to data obtained by Litke et al., 58% of outpatient clinics in Germany had their working hours reduced, and 15% had to be temporarily closed [28].

Among health workers directly involved in the fight against the pandemic, an increased workload was observed.

In the light of current results, during the COVID-19 pandemic in Poland, the number of physiotherapists working more than 40 h a week increased by 9% points compared to the pre-pandemic period.

Similar conclusions were drawn by Almaghrabi et al. who had examined the experience of dealing with the coronavirus pandemic among 1.036 medical workers in Saudi Arabia between March and April 2020. In the study group, 74% of employees agreed to work overtime [29]. A survey of nursing staff in England conducted by Rachel King found that 53% of nurses also experienced an increased workload during the pandemic [30].

The results of Misa Tomono’s observations concerning 4388 Japanese workers showed that excessive work, which was a particularly significant problem during the COVID-19 pandemic, could negatively affect the mental health of employees and reduce social interaction among them. Particularly strong correlations were observed among individuals running single-person households [31].

Changes in the professional activity of physiotherapists were mostly observed in countries particularly affected by the COVID-19 pandemic, such as Spain and Italy [32, 33]. The closure of rehabilitation wards and outpatient rehabilitation services in Spain during the first wave of the pandemic resulted in the redistribution of physiotherapists to other units. Many of them were delegated to work directly with COVID-19 patients, without prior training in this field. Following the closure of rehabilitation services, physiotherapists sought to continue their work with patients in the form of telerehabilitation (through videoconferencing and video instruction) [32]. In Italy, as a result of excessive overload of intensive care units, the work of physiotherapists providing pulmonary rehabilitation services was reorganized. The new duties of physiotherapists included, among others, monitoring oxygen therapy, conducting non-invasive ventilation of patients and postural therapy to improve oxygenation [33].

In the face of the COVID-19 pandemic, people began to change their health behaviours, including physical activity. The majority of the study participants reported engaging in high- to moderate-intensity recreational activities at least once a week, with men spending on average more than twice as much time as women. During the COVID-19 pandemic, the study subjects spent an average of three hours sitting or lying on a typical day, with higher results recorded in the group of women studied. There was also a significant increase in the duration of sedentary activity [34].

As shown by an online assessment of physical activity of physiotherapy students and physiotherapists, carried out by Srivastav et al., during the lockdown in March and April 2020, moderate to severe physical activity, as well as mobility and energy expenditure among the respondents reduced significantly as compared to the pre-lockdown period. There was also a significant increase in the duration of sedentary activity [34]. Similar findings were obtained by Mota et al., who studied changes in eating habits, physical activity and sleep during the COVID-19 pandemic among Brazilian healthcare workers (with physiotherapists accounting for 11% of the whole group of the respondents). It was found that nearly 54% of the study participants ceased regular exercise, 26% reduced the frequency of training, whereas almost 10% increased it [35]. In the Jadhav survey of Indian physiotherapists active during the pandemic, 70% of the respondents followed the WHO’s recommendations on the length of a week of moderate physical activity, while 30% devoted less than the advised 150 min per week to such activity [36]. Among the inhabitants of Italy, there was an increase in the number of people training five or more days a week from 6% before the pandemic to 16%. However, there was no increase in activity among people who did not exercise before the pandemic [37]. There was also a significant increase in the duration of sedentary activity [34].

The participants in our study were characterized in terms of eating habits during the COVID-19 pandemic. Most of the physiotherapists declared eating three or four meals a day. Similar results were obtained by Sidor and Rzymski, who studied the impact of the pandemic on the dietary choices and habits of the Polish population [38]. It was observed that more than half of the respondents did not change their intake of whole grains, dairy products, vegetables or fruits. An unfavourable increase was observed in the consumption of sweets, confectionery, salty snacks, and fast food products. (increase in the consumption of fast food products was also observed in over 25%, and a decrease in almost 30% of Italian respondents [37]. The Sidor study showed that nearly one third of the Polish population do not eat fresh vegetables or fruits every day, while one third declare that they consume sweets every day. Additionally, more frequent consumption of meat was observed in the group of men [38].

In contrast, in their study Di Renzo et al. showed that 34% of the surveyed Italian residents had an increased appetite during the lockdown period [37], which was accompanied by a higher consumption of homemade products (sweets, pizza, bread), legumes, white meat and hot drinks, and a reduction in the consumption of fresh fish, commercial sweets and pastries, as well as delivered food [38].

In the present study, it was observed that only a small proportion of physiotherapists started/ returned to smoking during the pandemic and/or smoked more cigarettes than before the pandemic. Similar results were obtained by Di Renzo et al., who observed a reduction in the number of people smoking more than ten cigarettes per day by 0.5% and smoking cessation in 3% than smokers. However, the survey conducted by Sidor et al. among the Polish population during the lockdown, does not support the abovementioned conclusions, showing an increase in the frequency of smoking in more than the 45% respondents [38].

There are numerous reports on increased alcohol consumption during the COVID-19 pandemic in the available literature [38], although our results showed that the majority of the participants in the self-study had remained at pre-pandemic levels. An upward trend was recorded, among others, in the group of Brazilian health workers in whom the consumption of alcoholic beverages, mainly beer and wine, increased by 27% during the COVID-19 pandemic [35]. A study published in Psychiatry Research indicates a link between higher alcohol consumption and younger age, male sex, and primary job loss due to COVID [39]. Moreover, according to the observations of Chodkiewicz et al., people who increased their alcohol consumption during the pandemic had also abused alcohol before [40]. According to McFarlane, experiencing prolonged or repeated exposure to one or more traumatic events can affect alcohol abuse. Ping Wu et al. examined the level of alcohol consumption among professionally active employees of a hospital in Beijing, three years after the outbreak of SARS-CoV1 in 2003. They showed that being in quarantine and working in a place with an increased risk of disease were significantly associated with subsequent symptoms of alcohol dependence or abuse [41].

In our study, we observed that the majority of the respondents slept from seven to eight hours a day on weekdays, and less than half slept less than seven hours. The results obtained by Stewart et al. showed that the average sleep time of healthcare staff working on the front lines during the COVID-19 pandemic was 6.1 h per day [42], including 7.04 h per day among nurses [43]. At the same time, insufficient sleep quality was reported by 95.5% of the respondents, whereas moderate to severe insomnia was found in 33% of the study participants [42].

Among Brazilian healthcare workers, 30% reported moderate difficulty falling asleep and 42% dissatisfaction with their current sleep patterns [35]. The results of a meta-analysis conducted by Salari et al. indicate that the prevalence of sleep disorders among nurses working with COVID-19 patients was 35%, and among doctors working with COVID-19 patients, it was almost 42% [44].

In our study, a much lower percentage of the respondents reported regular problems with falling asleep (over 10%). Due to sleep problems, 9% of the self-study respondents reported to have started taking sedatives during the pandemic, while 29% of healthcare workers in Brazil reported taking antidepressants, hypnotics, herbal medicines and melatonin [38].

The new epidemiological situation that occurred with the global spread of COVID-19, and the resultant risks and changes in the nature of work affected the level of stress and anxiety among healthcare workers. Stress levels among medical professionals during the COVID-19 pandemic were most significantly influenced by factors such as the rapid spread of COVID-19, insufficient knowledge about the disease, lack of personal protective equipment, concerns about new professional tasks, changes in family and social life, concerns about one’s own health and fear of transmitting the infection to others, as well as excessive workload [45]. As shown in the study by Zerbini et al., psychosocial support from family and friends, along with leisure time, were found to be the most important resources for doctors and nurses working with patients suffering from COVID-19 [46].

The statistically significant correlation was observed between the sex of the respondents and the level of perceived fear for the health of their loved ones, both in our own study and in the observation of Meo SA et al., who analyzed the level of anxiety, ranging from moderate to severe, and found it in 31.7% of women and 15.4% of men [47]. The researchers confirmed the increased risk of stress, anxiety and depression during the COVID-19 pandemic among healthcare workers. Nearly 50% of the subjects reported clinically significant anxiety, and the number of scores above the GAD-7 anxiety scale tripled during the pandemic. The results show a greater susceptibility to depression in the group of the studied women [48]. Zerbini et al., who surveyed 75 nurses and 35 doctors at a German hospital in Augsburg, noticed that nurses working directly with COVID-19 patients reported greater exhaustion, more stress and more depressive symptoms compared to those working in regular wards. Doctors working in COVID-19 wards did not report excessive mental strain, which may be related to shorter contact with COVID-19 patients compared to nurses. The participants of the study most often declared excessive workload and uncertainty about their professional future as the most common causes of psychosocial burdens [46].

### Strengths and limitations

This paper contains extremely important data in the epidemiological and social context, providing the basis for planning long-term prevention in the field of pro-health behaviours in the studied group. The results of the work can form grounds for preparation of preventive projects related to the protection of health of medical workers, especially in difficult epidemic conditions.

Despite the large number of studies on physiotherapy and physiotherapists during the COVID-19 pandemic conducted on global scale, and including limitations, therapeutic challenges, remedial actions, the impact of the COVID-19 pandemic on the health behaviours of professionally active physiotherapists has not yet been fully investigated. Research papers characterising active physiotherapists in this period are more focused on types of physiotherapy services in the face of the pandemic and physiotherapy management for COVID-19 in the acute hospital setting and beyond [49, 50]. Although the experience of the pandemic has shown a greater recognition for physiotherapy services worldwide [13], there are no reports on characteristic of healthy lifestyles among physiotherapists. Additionally, most of available observations do not provide information on how the COVID-19 pandemic affected health behaviours among professionally active physiotherapists. The results of research in this area are limited to students of medical faculties and healthcare workers’ physical activity, diet, and well-being during the COVID-19 pandemic [51]. The direction of future research is to determine the health behaviour of healthcare workers, including physiotherapists, following the lockdown period and lifting of the epidemic emergency state. The obtained results confirmed the need to modify health behaviours in the group of physiotherapists examined during the COVID-19 pandemic, which could reduce adverse changes in professional life and negative attitudes. Moreover, they are a valuable signpost for the future, in the case of any other potential pandemic.

We are aware that the study also has some limitations. One of them was the lack of data on the respondents’ physical activity and other health behaviours before the COVID-19 pandemic, which would undoubtedly be useful for a more accurate analysis, allowing a comparison of the examined indicators before and during the pandemic. Another limitation is the fact that the study group was divided by gender, and the relationship between the percentage of males and females was not equal (71% vs. 29%, respectively), which may restrict the full interpretation of the data. Additionally, it should be emphasized that physical activity in our respondents was measured by a questionnaire, which is a subjective method of assessment, and therefore its validity and reliability may be questionable. Moreover, self-reported physical activity is often overestimated. Still, a questionnaire is one of practical methods for evaluating this parameter among subjects, more so that it contains questions from a standardized questionnaire. However, our observations also have considerable advantages over the other available analyses since as one of a few, it comprehensively characterizes the specificity of work and health behaviours of physiotherapists during the COVID-19 pandemic.

## Conclusions

The COVID-19 pandemic caused changes in health behaviours in some of the surveyed physiotherapists. In that period, the average weekly working time of professionally active physiotherapists increased. Also, the time physiotherapists devoted to physical activity was extended - to 240 min per week for low- and moderate-intensity activities. It was found that during the pandemic, physiotherapists more frequently made nutritional errors including increased calorie intake and consumption of sweets, confectionery, fast food products, alcohol, coffee and energy drinks. Additionally, an increased frequency of smoking was also observed. Almost 10% of all the physiotherapists started using pharmacological sedatives. During the pandemic, this professional group was exposed to higher levels of stress related to, among others, the risk of contracting COVID-19, and concerns about the health of their loved ones or job loss. At that time, they also experienced sleep problems that had not occurred before.

Further studies are required to assess whether physiotherapists’ health behaviours returned to baseline levels or slightly improved compared to the initial results. Also, it is necessary to introduce health-promoting initiatives that would focus on physiotherapists, support their positive health behaviours and provide special recommendations helping them to maintain health during a pandemic.

## Data Availability

The datasets used and/or analysed during the current study available from the corresponding author on reasonable request.
